# Understanding Different Types of Recreational Runners and How They Use Running-Related Technology

**DOI:** 10.3390/ijerph17072276

**Published:** 2020-03-27

**Authors:** Mark Janssen, Ruben Walravens, Erik Thibaut, Jeroen Scheerder, Aarnout Brombacher, Steven Vos

**Affiliations:** 1 Department of Industrial Design, Eindhoven University of Technology, 5612 AZ Eindhoven, The Netherlands; a.c.brombacher@tue.nl (A.B.); s.vos@tue.nl (S.V.); 2 School of Sport Studies, Fontys University of Applied Science, 5612 AR Eindhoven, The Netherlands; r.walravens@fontys.nl; 3 Policy in Sports & Physical Activity Research Group, University of Leuven, 3000 Leuven, Belgium; erik.thibaut@kuleuven.be (E.T.); jeroen.scheerder@kuleuven.be (J.S.)

**Keywords:** recreational running, typology, sports watches, mobile applications, clusters, attitudes, interest, running-related technology, wearable

## Abstract

This study aims to help professionals in the field of running and running-related technology (i.e., sports watches and smartphone applications) to address the needs of runners. It investigates the various runner types—in terms of their attitudes, interests, and opinions (AIOs) with regard to running—and studies how they differ in the technology they use. Data used in this study were drawn from the standardized online Eindhoven Running Survey 2016 (ERS2016). In total, 3723 participants completed the questionnaire. Principal component analysis and cluster analysis were used to identify the different running types, and crosstabs obtained insights into the use of technology between different typologies. Based on the AIOs, four distinct runner types were identified: casual individual, social competitive, individual competitive, and devoted runners. Subsequently, we related the types to their use of sports watches and apps. Our results show a difference in the kinds of technology used by different runner types. Differentiation between types of runners can be useful for health professionals, policymakers involved in public health, engineers, and trainers or coaches to adapt their services to specific segments, in order to make use of the full potential of running-related systems to support runners to stay active and injury-free and contribute to a healthy lifestyle.

## 1. Introduction

Public health is an important policy goal for policymakers [1], since the number of people with lifestyle-related health problems has increased [2]. Fortunately, being physically active contributes to a healthy life and reduces the risk of chronic diseases [3]. Running is a popular form of physical activity that contributes to a healthy lifestyle. Moreover, running is one of the most popular exercise activities in the world in terms of participation [4,5], and is practiced by a diverse and heterogeneous group of people [6,7,8,9]. The popularity of running is probably due to its health-related benefits (i.e., musculoskeletal and cardiovascular health, body composition, and psychological state) [10,11], and low entry-level. It is relatively inexpensive and easy to practice [12,13]. 

Unfortunately, dropout rates are high due to running-related injuries and demotivation [14,15,16,17,18,19,20,21]. In line with the growing dropout rates, in recent years there has been an exponential increase in the availability and use of sports and physical activity-related monitoring devices such as mobile applications (apps), watches, and activity trackers, which claim to support runners [22,23,24,25]. These sports watches and apps have great potential, because they are affordable, accessible, have a large reach, and provide multi-function operation [26,27,28], but the literature shows that this kind of technology is often not used for prolonged times and user commitment is low [29,30,31]. Therefore, it is important to match the user’s expectations of technology and close the gap between runners’ expectations and actual experience with these devices [6,32,33]. In order to understand the needs of runners effectively and adequately, their attitudes, interests, and opinions (AIOs) should be established and understood. Various researchers [34,35,36] have shown that AIOs are essential in understanding users’ habits. However, studies [25,37,38] exploring running-related technology have only focused on the relationship with demographic and running-related variables. 

### 1.1. Running-Related Technology

Inspired by the quantified self-movement, an increasing number of people are using technology to monitor themselves [39,40,41]. Pobiruchin et al. [37] showed that about 75% of runners used wearable technology for training optimization and distance recording, and provided insights into the large variety of wearable and smart technology in use. A study by Janssen et al. [6] revealed that recreational runners differed significantly in the technology they used for their sport; 60% used a sports watch and more than half (53.3%) used dedicated apps. Clermont et al. [25] found that among runners, tracking personalized training data was the main reason for using technology, which suggests that the biggest motivators are instant feedback (rewards for achieving targets) and insight into achievements (e.g., distance covered, average speed). Not only has the number of runners using technology increased, but the development of systems related to running (and other sports) has received significant attention in research. Although researchers face real challenges in using technological systems to prevent running injuries [42,43], several studies focus on aspects of improving running technique and minimizing injury [44,45,46,47]. Other researchers focus more on the role of running technology in relation to the social aspects of running [48,49,50,51] and the individual motivation of runners [52,53]. 

### 1.2. Different Types of Runners

Given the heterogeneity among runners, segmenting them into groups in order to understand their AOIs is useful and appealing. Segmentation of consumers in sports has been documented extensively (e.g., [54,55,56,57]), with studies typically differentiating between consumers based on demographic factors. This traditional form of demographic segmentation is used because researchers apply the concept that gender and age can influence running preferences. The findings of Ogles and Masters [34] support this notion, as age significantly differed among their groups. However, they also found that different types of marathon runners were distinguishable by not only their demographic characteristics, but also their behavioral and psychographic variables. Other studies [35,58,59] also used psychographic variables, including AIOs, to cluster runners. 

Variables such as health, runner identity, personal goal achievement, the social aspects of running, running addiction, commitment, competition, and ease of practice have been used to segment runners [6,35,58,59,60,61,62]. To gain a better understanding, for example, Parra-Camacho et al. [60,61] segmented runners according to commitment and reasons to partake in running. Rohm et al. [35] showed that a group they referred to as social competitors scored high on motives like competition and social reasons, while Vos et al. [58] found two groups of social runners: one group that scored high on both social and competition motives, and a group they called “companion runners”, who scored high on social motives and low on competition. A study by Forsberg [59] showed that runners with less running experience (≤3 years) focused more on AIOs related to health, whereas more experienced runners (≥8 years) were more likely to run “for the love of running” or for social reasons. 

Finally, all of the studies mentioned above [6,34,35,58,59] stress the importance of AIOs in gaining valuable insights into the needs and requirements of runners. These AIOs provide an effective basis for segmenting runners and creating runner typologies. 

### 1.3. Aim of the Study

Studies have found that running-related technology such as sports watches and apps are widely used, and they get significant attention in research. However, consumer-available running apps and sports watches almost all use the one-size-fits-all principle and take little or no account of a runner’s motives, drivers, or AIOs [6,32,63]. These insights suggest a need for a more differentiated approach that targets the distinct needs of specific types of runners [6,28].

Several studies [6,34,35,58,59] have shown that AIOs are important and can provide valuable insights into the behavior of runners. To the best of our knowledge, none of the existing studies provide insights into the use of running-related apps and sports watches in relation to runner AIOs. This study is a follow-up of previous research (Janssen et al. [6]), which gave the first insight into the characteristics of runners using apps and sports watches and proposed an approach to estimate their use based on runners’ characteristics. With the present study, we sought more in-depth insight into AIOs of runners. This study aims to (i) investigate how AIOs regarding running combine into distinct runner profiles, and (ii) show similarities and differences between these runner profiles in the use of running-related technology.

## 2. Materials and Methods 

### 2.1. Study Design and Respondents

In this study, an online questionnaire, the Eindhoven Running Survey 2016 (ERS2016), was used to collect data among participants of the Eindhoven Marathon running event. This event consisted of 4 running distances (42.2 km, 21.1 km, 10 km, and 5 km). Survey questions were derived from a standardized questionnaire used in previous occurrences of this event (ERS2014 [6] and ERS2015 [64]). 

After completion of the event, all registered participants (N = 18,261) received an email with an introductory letter and a web link to the online questionnaire. All participants agreed to be contacted for research purposes after registration. The introductory letter gave them information on the purpose of the study, allowed them to give informed consent, and guaranteed that their data would be processed anonymously. The research conducted was in line with the ethical principles of the Declaration of Helsinki and the American Psychological Association [65]. The privacy of all participants was guaranteed, and all data were anonymized before analysis. The Research Board of the Fontys School of Sport Studies was consulted prior to initiation of this study, and approval for the study design was obtained. 

A total of 3727 participants fully completed the questionnaire (response rate of 20.4%), out of which 20.9% had participated in the marathon, 55.1% in the half marathon, 16.2% in the 10 km run, and 7.9% in the 5 km. The average age of the respondents was 42.2 years, and their ages ranged between 18 and 81 years old. Approximately one-third of the participants were women (33.2%), approximately 9 out of 10 participants were employed (89.9%), and 71.4% had received a higher education (a summary of the sample is represented in stacked column graphs in Figure S1, or see Table 3, last column). The sociodemographic characteristics of the respondents were comparable to samples used in prior large-scale running studies conducted in western Europe [4,6,64]. 

### 2.2. Questionnaire

**Figure 1:**
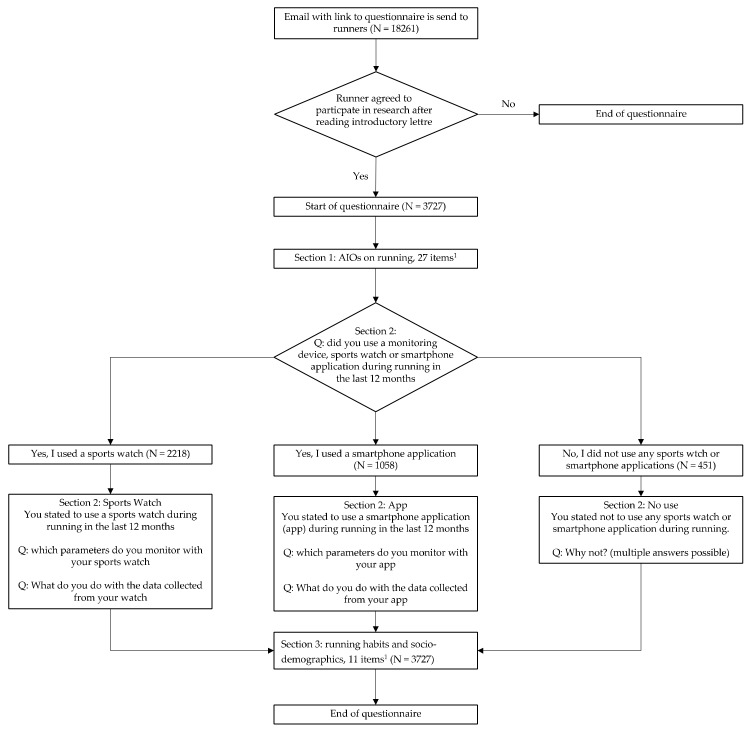
Flowchart of ERS2016 questionnaire including number of respondents. ^1^ For details of the items see Supplementary File S1, Questionnaire ERS2016.

The online questionnaire had 3 sections, covering (i) AIOs on running, (ii) the use of running-associated technology, and (iii) sociodemographics and running habits. The questionnaire is provided in the Supplementary Materials (File S1, questionnaire ERS2016), and Figure 1 shows a flowchart of the questionnaire.

The first section of the questionnaire consists of 27 items, with 25 questions on running AIOs, and was adopted from previous studies [6,34,35,58]. We asked the respondents to rate the extent to which they agreed with the items, using a 5-point Likert scale (1 = totally disagree, to 5 = totally agree). Items included assertions such as “I can practice running anytime”, “Running gives me energy”, and “I am proud to be a runner”. There were also items such as “I would quit running if I got injured” and “I would quit running if my trainer quits”. The present study included two additional items related to competitiveness: “Running is a competitive sport” and “Running is a performance sport”, since we also wanted to gain insight into possible AIOs regarding competitiveness in running. This section included a total of 27 scored items.

The second section of the questionnaire collected insights into the use of technology by runners. First, respondents were asked whether they used technology while running, and if so, what they used most frequently (no use/use of app/use of sports watch). Then the questionnaire progressed to items specific to non-users, app users, or sports watch users. Questions included “What data do you monitor while running (distance, time, speed, heart rate, other)” and “What do you do with the monitored data (nothing, review the session after a run, monitor data over time, or use the data to adapt future training)”. Non-users were asked for their reasons for not using technology (“running with phone/watch is ignorant”, “no added value”, “no need to”, “does not fit my running needs”), with the responses recorded using the Likert scale.

The third and final section of the questionnaire covered sociodemographic characteristics, including gender (male/female), age, professional status (student/employed full-time/employed part-time/unemployed), and level of education (lower and middle/higher/university). The aspects that were covered regarding running habits included running distance (5 km, 10 km, 21.1 km, 42.2 km); most practiced sport (running/other sport); years of running experience (<1 year: novice; 1–5 years: moderately experienced; >5 years: experienced); running distance (average per session); running frequency (number of runs per week); event participation (number of running events participated in over the last year); and running context (individual, with friends, colleagues, and/or running groups, or clubs). 

### 2.3. Analysis

#### 2.3.1. Typology Construction 

**Figure 2:**
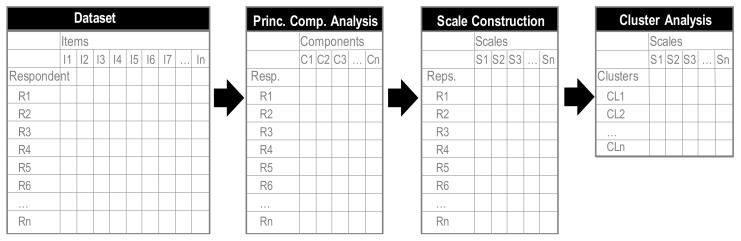
Series of analyses to construct typology: (i) principal component analysis (PCA) with orthogonal varimax rotation including Cronbach alpha reliability analysis; (ii) scale construction by calculating average scores for reliable items per component; and (iii) K-means cluster analysis.

To construct the typology, a series of analyses were conducted using SPSS 25.0 (see Figure 2). First, to reduce the 27 AIO items to components, principal component analysis (PCA) with orthogonal varimax rotation was executed. In PCA, one of the most commonly used criteria for solving the number of components aspect is the eigenvalue-one criterion [66,67]. We applied this approach by including all components with an eigenvalue of >1.00, by which the components were assessed on the content of the included items. Second, a reliability analysis was executed for all components, with Cronbach α scores of >0.700 considered acceptable, by which items were assessed and reconsidered if they substantively contributed to the component. Then, scales were constructed by calculating the average scores for the reliable items per component, resulting in average scale scores. Finally, in order to create the typology, a K-means cluster analysis was performed using the constructed scales. 

The K-means cluster analysis technique was chosen because the data involved a high number of cases [68], and it was the optimal method considering both within-cluster and between-cluster heterogeneity [69]. This type of cluster analysis was applied in previous segmentation studies [35,70,71,72,73]. Solutions between 2 and 6 clusters were checked and assessed on the basis of variability and heterogeneity. Through crosstabs, including chi-square with Bonferroni corrections, we checked whether the clusters were significantly different from each other on the scales, and iteration history and distances between cluster centroids were checked, looking for the solution with the least iterations and largest distance between cluster centers. Clusters were also checked on the distribution of numbers of runners across the clusters, and finally, clusters were assessed content-wise on practical applicability.

#### 2.3.2. Crosstabs 

In order to obtain insights into the use of technology (including what data are being monitored and how the monitored data are being used) between different typologies, crosstabs, including chi-square tests with Bonferroni corrections, were used. 

## 3. Results

### 3.1. Runner Profiles 

#### 3.1.1. Principal Component Analysis

PCA (eigenvalue > 1.00) resulted in six components, accounting for 61.73% of the variance. Five items scored PCA coefficients of <0.30 and also loaded on multiple components. Based on these PCA coefficients and item content, we decided that none of the five items suited any of the six components. Therefore, these five items were removed, and the remaining 22 items were included in the next analysis. The six components, and some examples of the items included in them, are as follows:

1. Perceived advantages of running (e.g., “running gives me energy” or “running is good for my health”);
2. Social motives for quitting (e.g., “I would quit running if my trainer quit” or “if my running friends quit”);
3. Identification with running (e.g., “I am proud to be a runner” or “I consider myself to be a real runner”);
4. Running is a sport that is easy to practice (e.g., “I can practice running anytime, anywhere”);
5. Individual motives for quitting (e.g., “I would quit running if I got injured” or “if my spare time was decreased”); and
6. Competitiveness in running (e.g., “running is a competitive sport” or “running is a performance sport”).

The reliability analysis revealed that these six components achieved Cronbach α values ranging from 0.697 to 0.935. One item of component 2 was removed, based on the content of that item (Cronbach α range then increased to 0.848–0.935). Component 6 consisted of just two items, as we only added two items on competitiveness to the existing questionnaire. The scores for the reliable items per component were averaged to make scales. Table 1 shows the six scales, including number of items, Cronbach α value, average score, and standard deviation.

#### 3.1.2. Cluster Analysis

K-means solutions for two to six clusters were assessed. Clustering the dataset into four clusters proved to be the most suitable solution considering iteration history, distance between cluster centroids, and equal runner distribution across the clusters. Solutions with two or three groups did not account for the heterogeneity of runners across clusters (i.e., small distance between cluster centers), resulting in clusters with only very high or very low scores on all scales. Solutions of four, five, and six clusters accounted for runner heterogeneity, although clustering into five or six groups resulted in highly unequal group distribution, including two very small groups, with N < 25. Therefore, eventually, four clusters were considered the most suitable solution.

The results of the analysis show 886 type I runners (23.8%), 1008 type II runners (27.0%), 1012 type III runners (27.2%), and 821 type IV runners (22.0%). In Table 2, the results of chi-square tests (with Bonferroni corrections) show that the four types differ significantly across all six scales. 

### 3.2. Characteristics of the Typology

Based on their AIOs with regard to running, four types of runners were identified: (i) casual individual (type I), (ii) social competitive (type II), (iii) individual competitive (type III), and (iv) devoted (type IV). 

#### 3.2.1. Type I: Casual Individual Runners

Compared to other types, type I runners identified with running the least and were the most susceptible to quitting the sport for individual reasons, thus they also scored low on competitiveness (see Table 2). Type I runners were classified as casual individual runners, and the sociodemographics showed that this group consisted of relatively more women, runners <35 years of age, runners with higher education, and students compared with the other types. Considering the habits of runners, the analysis showed that this group comprised relatively more 5 km and 10 km runners, more runners for whom running was not their main sport, more inexperienced runners, and more runners who trained less frequently, participated in fewer events than others, and ran more individually compared to other types. 

#### 3.2.2. Type II: Social Competitive Runners

Type II runners were characterized as competitive and were the most susceptible to quitting in general, especially for social reasons (see Table 2). We referred to them as social competitive runners, and this was not a group that stood out (scoring highest or lowest of all types) in terms of sociodemographic. An analysis of their running habits showed that the type II group included relatively more 5 km and 10 km runners (as noted for type I). The social competitive runners group scored relatively higher (compared to individual competitive and devoted runners) with regard to runners for whom running was not their main sport, less experienced runners, and runners who trained less frequently and participated in fewer events than others, while casual individual runners scored even higher on these items. For running context, social competitive runners scored the lowest on running individually, but had the highest scores for running with friends, colleagues, small groups, and clubs. 

#### 3.2.3. Type III: Individual Competitive Runners

Type III runners were classified as individual competitive runners and were characterized by their competitiveness, and their lower susceptibility to quitting either as individuals or socially (see Table 2). In contrast to the previous group, they scored well on aspects such as perceived advantages of running and identification with running. The distribution of gender within this group differed the most compared to other groups, with the highest proportion of male runners out of all four types. The group also had the most participants with lower or middle education and the lowest number of students. 

With regard to running habits, individual competitive runners scored high on running as the main sport, long training distances, frequent training sessions, and participating in five or more events annually. While these habits did not differ from devoted runners, the individual competitive group ran individually more than either devoted or social competitive runners. 

#### 3.2.4. Type IV: Devoted Runners

Similar to type III, type IV runners scored high on the perceived advantages of running and identification with running and had low susceptibility to quitting (either as individuals or socially) but were not as competitive as other types (see Table 2). We, therefore, called them devoted runners, and among the groups, they included the most runners older than 45, the most with low or middle education, and the most employed part-time. This group scored high on running as the main sport, long training distances, frequent training sessions, and participating in five or more annual events, similar to type III. Devoted runners are the most experienced and, with social competitive runners, had the most club runners. Some background characteristics applicable to the running groups are listed in Table 3 (for stacked column graphs of Table 3 see Figures S1–S5).

### 3.3. Use of Apps and Sports Watches

Descriptive analysis revealed that among the 3727 runners, 6 out of 10 used a sports watch (59.5%) and almost one-third used an app (28.4%), with the remainder (12.1%) using neither. Next, data monitoring and what runners do with the data were analyzed for app users (N = 1058), sports watch users (N = 2218), and non-users (N = 451).

Almost all app users monitored distance (98.2%,), time (96.6%), and speed (94.2%). A minority monitored heart rate (9.1%) or other parameters such as cadence or kcal (5.4%). Eighty percent of the app users (80.3%) used the data to review the session afterwards. Approximately 60% of all app users (56.9%) also monitored data over time. One in 10 app users (11.7%) actually used the data to adapt their training, and 6.4% did nothing with the monitored data. 

Among the sports watch users a similar tendency is shown; most of the users monitored distance (96.0%), time (90.0%), and speed (85.5), and a small group monitored other parameters such as cadence or kcal (8.8%). For heart rate monitoring, however, there are differences between sports watch users and app users: 7 out of 10 sports watch users (68.2%) monitored their heart rate. Again, a minority of sports watch users (5.7%) indicated that they did not do anything with the monitored data. Almost 80% used the data to view the session afterwards (77.3%), 56.6% used the data to monitor over time, and 22.3% use the monitored data to adjust their workout.

For the group that did not use technology, they were asked for reasons why. The four main reasons they gave for not using technology while running were: (i) “running with phone/watch is ignorant” (33.8% of non-users), (ii) “using technology has no benefit” (40.2%), (iii) “there is no need to” (37.4%), and (iv) “using technology does not fit my running needs” (24.1%). 

### 3.4. Use of Apps and Sports Watches in Relation to Different Type of Runners

Crosstabs, including chi-square with Bonferroni correction, provided insight into the differences in technology used by different types of runners (Table 4). The results revealed significant variations (only significant effects are described). In relative terms, casual individual runners were the keenest app users (41.1%) and the smallest group of sports watch users (44.8%), while social competitive runners included fewer app users (26.7%) than casual individual (41.1%), about the same as individual competitive (25.3%), and more than devoted runners (20.7%). Social competitive runners included more sports watch users than the casual individual group (57.8% vs. 44.8%), and less than either individual competitive (68.0%) or devoted (67.1%) groups. The lowest proportion of non-users (6.7%) was found among the individual competitive runners compared to the other types (12.2%, 14.1%, and 15.5%), while they and the devoted runners had the highest uptake of sports watches (68.0% and 67.1%, respectively). Finally, the devoted group had the fewest app users (20.7%), and together with the individual competitive runners had the highest proportion of sports watch users. 

Table 5 shows the comparison between the types of runners and the data they monitored with an app and what they did with the data. Among app users, significant differences were found in the data they monitored, such as cadence and energy use (kcal). Devoted runners monitored “other” data more than casual individual runners (9.4% vs. 3.0%). In terms of data usage, a difference was seen in the use of monitoring data over time, as fewer casual individual (53.6%) and social competitive (54.3%) runners monitored their data over time, compared to individual competitive (65.6%). No differences were found in relation to the other data items, “to review the session after a run” or “to adapt training”. 

In terms of monitoring distance, speed, and heart rate, there are significant differences between types of runners (Table 6). Individual competitive runners scored highest on monitoring distance (92.3%) and speed (91.0%). Although devoted (91.3% and 88.4%) and social competitive (88.0% and 87.3%) runners also made extensive use of the data for heart rate monitoring, social competitive runners scored lower than individual competitive runners (65.2% vs. 72.1%), with the casual individual group recording 68.8%. Looking into data usage, more differences were found. On doing nothing with the data (*p* = 0.059), individual competitive (4.2%) and devoted (4.9%) runners scored lower than casual individual (6.8%) and social competitive (7.4%) runners. On monitoring data over time and using the data to adapt training, individual competitive runners (65.3% and 29.7%, respectively) scored significantly higher than all other types (others <54.2% and <22.0%, respectively). No differences were found in the data item “review the session after a run”.

Regarding the four main reasons provided for not using technology, two showed significant differences between two types of runners. The reason “technology has no added value” scored lower among social competitive runners than devoted runners (32.1% versus 51.5%), and the same for “does not fit my running needs”, at 17.3% vs. 32%, respectively (see Table 7).

## 4. Discussion

Running is one of the most popular exercise activities in the world [4,5], and is known for its health benefits [10,11]. Unfortunately, running also has high dropout rates due to injuries and demotivation [14,15,16,17,18,19,20]. Running-related technology such as sports watches and apps have great potential to support runners in their training activities [26,27], guide them in running injury-free, and motivate them. Yet, the literature shows that this kind of technology is often not used for prolonged times [29,30,31]. In order to better exploit the potential of running-related technology to support runners, a better understanding of runners is necessary. Therefore, we constructed a typology based on their stated running AOIs and analyzed whether different types of runners differed in their use of sports watches and apps.

Based on a series of statistical analyses, four runner types were identified. The clusters met the criteria for relevant and valuable segments as stated by Kotler et al. [74]. They were measurable in terms of number of runners per segment, had significant volume, and were differentiable, since they differed substantially from each other. 

The constructed typology showed similarities with previous research. For example, Parra-Camacho et al. [60] segmented runners based on reasons to partake in running. Their so-called individual hedonists showed similarities to our casual individual and individual competitive runners. These groups experience running as an individual activity. The two types of individual runners in our study differ in the extent to which they are competitive and their identification with other runners.

The group of dedicated runners consists of experienced long-distance runners who identify strongly with running. This group is similar to other types of runners from previous research, notably devotees, as described by Rohm et al. [35], running enthusiasts, by Ogles and Masters [34], and enthusiasts by Parra-Camacho et al. [60]. Our individual competitive runners showed some similarities to the personal goal achievers and personal accomplishers of [34], and individual runners in Vos et al. [58], although our type seemed to be more competitive, as opposed to the more health-focused types of Ogles and Masters [34] and Vos et al. [58]. 

The type that Rohm et al. [35] called social competitors, runners who scored highly on motives such as competition and social reasons, and the competitive achievers from Ogles and Masters [34] were comparable to our social competitive runners. These runners were characterized by AIOs related to competitiveness and social motives. 

In contrast to Vos et al. [58], who found two groups of social runners, one scoring high and one low on competition, we only found one group of social runners; this outcome could be due to the differences in the sample, as Vos et al. [58] investigated a women-only event. They argued that due to the more homogeneous nature of their sample, other types of runners were found. 

Finally, our casual individual runners, who were characterized by low identification with running and did not consider running their main sport, had no equivalent in the previous literature. Our ability to differentiate this group in our research can perhaps be explained by the popularity of running. Partly as a result of the increased number of events, more people are running for whom running is not their main sport and who have limited identification with other runners. 

In conclusion, we managed to segment a heterogeneous group of runners into four smaller homogeneous groups, thereby providing valuable insights into the AIOs of the different groups in order to differentiate between types of runners and focus on their potential interests.

### 4.2. Use of Running-Related Technology among Runners

Research showed that technological devices are popular among runners [6,25,37]. This holds true for the current study, as 87.9% of the participants used a technological device, either an app or a sports watch, although we found fewer app users among runners (28.4% in the current study, as opposed to >50% in other studies), compared to Clermont et al. [25] and Janssen et al. [6]. We argue that this lower number was due to the differences in the sample and the types of questions asked, as we included a broader range of runners, from beginners to very experienced, and from 5 km to full marathon runners. Our questionnaire also required respondents to identify their most frequently used technological device, whereas in Clermont et al. [25] and Janssen et al. [6], runners could choose multiple answers, with the option of answering that they used both a sports watch and an app (e.g., Garmin sports watch with compatible Garmin Connect app). This probably increased the reported quantity of app usage, whereas in the present study, the runners were classified by their first choice, in this example as sports watch users. 

We found that runner types differed in the kind of running-related technology they used. Casual individual runners made up the largest group of app users; it included younger, less experienced runners, and more recreational runners. Both Clermont et al. [25] and Janssen et al. [6] found that this particular group of novice and recreational runners used apps more often, perhaps because their lower commitment to running made them prefer lower-cost technology (apps) over more expensive sports watches. This concept was supported by other data, as it was found that more competitive and experienced runners (i.e., individual competitive and devoted) used sports watches more often, indicating that runners who identified with and were more involved in running were likelier to spend more than the others. 

When considering data monitored with the devices, for both apps and sports watches, GPS-based data (time, distance, and speed) were used by almost every runner. One difference between app and sports watch users was in their collection of heart rate data while running: among app users the take-up rate was <10%, while considerably more sports watch users (59.5%) collected heart rate data. The reason for this difference could be that apps do not measure heart rate, so app users need to buy another device (e.g., Bluetooth heart rate monitor) and connect it to the smartphone. This not only takes extra effort, but also monetary investment, while one of the advantages of using apps is that they are free (or inexpensive) to download. Meanwhile, sports watches are often equipped with a built-in heart rate monitor or sold as a package including a monitor. Given that sports watch users collected more objective training data such as heart rate, we expected that they would use the data differently from app users, and as expected, we found that twice as many sports watch users (22.3%) used the data to adapt their training compared to app users (11.7%). We expected this difference to be greater, since heart rate can be a useful measure by which to adapt training. However, we concluded that learning about heart rate, using heart rate information, and applying heart rate data might be too complicated for many runners. 

Regarding the different types of runners, we found that more individual competitive runners used a sports watch to monitor heart rate than social competitive runners did (72.1% vs. 65.2%, respectively). The individual competitive group also had the most runners who used this measure to monitor training over time (65.3%) and to adjust their training (29.7%). This finding is in line with the competitive nature of this type of runner, although it might also appeal to social competitive runners, given their similar competitive nature. We argue that the differences between individual competitive and social competitive runners in their scores on competitiveness in running (4.25 vs. 3.70) illustrate this. Such competitiveness is also reflected in more thorough monitoring of running performance and the use of the data to create the next training.

As mentioned earlier, tracking personalized training data has been cited as the main reason to use technology [25]. Several studies focus on designing new technology for runners to improve their running technique and prevent injury [44,45,46,47], to enhance the social interaction between runners [48,49,50,51], or to motivate individual runners [52,53]. Although technology seems to offer many advantages for runners, Hein et al. [75] state that there are also associated (societal) risks, such as loss of social cohesion, privacy, and awareness. In our study, we found a small group of non-users. They had various reasons for not using running-related technology, ranging from the practical, such as “running with a device is ignorant”, to how they want to be involved in running, such as “does not fit with my authentic running experience”. This in line with findings of Deelen et al. [64], who showed that some people run because they want to enjoy and experience their environment, and do not bother monitoring their performance. When considering the different type of runners, there were some differences in the reasons not to use technology. The reasons “it has no added value” and “it does not fit my running experience” were given more by devoted than social competitive runners. We suspect that the low scores on competitiveness might be related to a lower interest in running with technology to monitor data. Also, dedicated runners are the most experienced, so it is likely that they know their bodies well after years of training and do not need to monitor their performance through a monitoring device. To the best of our knowledge, this particular group of non-users and their motives have not yet been studied and could be an important topic for future research. 

Based on our results, we argue that the technology available on the market today is not yet targeted to specific segments or fails to target a specific segment. Our approach might help professionals to differentiate between types of end users, and to design for a specific target group. 

### 4.3. Limitations

Certain limitations can be highlighted. The sample size in our study did not allow all runners to contribute. Instead, a sub-sample focusing on event runners was selected, and the running event participants were considered to be a representative selection of the broader recreational running community [76]. Future research could consider different runner samples, in an effort to ensure representation of all potential runner types. We also believe that studying data from different countries would illustrate geographic variations in sports culture.

We included runners of various distances, ranging from the full marathon to the 5 km city runs, and this could be seen as a limitation, given the large range of running experiences. We believe that our sample reflected the apparent heterogeneity of runners, insofar as their AIOs were the main typology focus, rather than the distance covered or runner experience. 

Certain methodological limitations concerning the dependent variables are mentioned below. First, the intensity of device use and the reasons why a specific brand was used were not investigated when we asked runners to choose their most-used technological device. Focusing on the reasons why particular brands are used and what features runners are looking for could be beneficial for future research. Second, we did not ask what features on the watch or the app the runners used. It would be interesting to find out which features are used by different groups of runners and if there are different preferences for features that provide guidance and support. Third, we used only two items to measure AIOs on competitiveness in running, although they scored an acceptable Cronbach α of 0.697. Future research should consider replicating the current study with more items on this topic.

Finally, respondents who did not use running-related technology could only choose between four possible reasons why not. In future research, it might be interesting to look into this topic, using theories about technology acceptance and items on the benefits, risks, and consequences of technology use.

### 4.4. Implications

This study elaborated on a previous study [6] to better understand runners’ AIOs and the use of running-related technology. Our typology allows professionals working in the fields of public health, sports, and engineering to better understand their target groups. The differentiation between different types of runners can be used to adapt services to specific segments based on AIOs. Policymakers involved in public health may use the typology to specifically target particular runners and match their policies to the needs of runners. Trainers, coaches, and physiotherapists, for example, could support runners to match running-related technology with their AIOs

Finally, in the field of human–computer interaction, personas are in common use to give insight into the values, needs, user experiences, and interests of end users (see, e.g. [77,78,79]). This user-centered approach is an essential part of the design process, and a typology provides a solid basis to develop personas. Stragier and colleagues [80] advised that a segmented approach was preferable, in order to tailor app interfaces to user motivations; this segmented approach helps designers to differentiate between different end user types and their interests. This step is important, as many technological systems are still not reaching their target groups of product users [79,81,82,83], therefore the full potential of running-related systems that support runners to stay active and healthy has not yet been reached.

## 5. Conclusions

Our study shows that runner profiles based on AIOs can successfully differentiate the use of running-related technology (sports watches and apps) and give more in-depth insight into the needs and interests of runners. These insights into runner AIOs could help professionals in the field of running and running technology to provide value to end users. This, combined with the characteristics of different runner types, should help utilize the full potential of running-related systems to support runners to stay active and injury-free and contribute to a healthy lifestyle. 

## Figures and Tables

**Table 1:** Scales including number of items, Cronbach α, average score, and standard deviation.

Scale	Attitudes toward Running	Items	Cronbach α	N	Mean	SD
1	Perceived advantages of running	4	0.805	3666	4.35	0.479
2	Social motives for quitting	3	0.935	3700	1.65	0.731
3	Identification with running	5	0.787	3364	3.54	0.651
4	Running is a sport that is easy to practice	3	0.775	3709	4.24	0.625
5	Individual motives for quitting	4	0.716	3365	3.18	0.766
6	Competitiveness in running	2	0.697	3708	3.55	0.738

**Table 2:** Mean scores with standard deviation per type of runner for all six scales. Comparisons between types of runners via chi-square with Bonferroni adjustment.

Attitudes toward Running	Type I (N = 886)	Type II (N = 1008)	Type III (N = 1012)	Type IV (N = 821)
Perceived advantages of running	4.12 (0.48) *	4.18 (0.41) *	4.64 (0.39) **	4.43 (0.44) **
Social motives for quitting	1.46 (0.55) **	2.50 (0.58) **	1.26 (0.43) *	1.34 (0.47) *
Identification with running	2.88 (0.52) **	3.54 (0.50) **	3.98 (0.52) **	3.70 (0.50) **
Running is a sport that is easy to practice	4.16 (0.63) *	3.96 (0.62) **	4.53 (0.51) **	4.26 (0.58) *
Individual motives for quitting	3.76 (0.48) **	3.48 (0.52) **	2.90 (0.75) **	2.49 (0.57) **
Competitiveness in running	3.12 (0.64) **	3.70 (0.51) **	4.25 (0.45) **	2.98 (0.54) **

** *p* < 0.001; * *p* < 0.01.

**Table 3:** Independent variables related to type of runner in percentages, tested with chi-square and Bonferroni adjustment between types.

Variable	Measurement	Type of Runner				Average
		Casual Individual	Social Competitive	Individual Competitive	Devoted	
Gender	Male	64.5 ^a^	66.2 ^a,b^	71.3 ^b^	63.3 ^a^	66.8
	Female	35.5 ^a^	33.8 ^a,b^	28.7 ^b^	36.7 ^a^	33.2
Age	≤35 years	38.6 ^a^	29.7 ^b^	24.0 ^c^	15.3 ^d^	27.1
	36–45 years	33.5 ^a^	30.3 ^a^	34.5 ^a^	30.0 ^a^	31.9
	≥46 years	27.0 ^a^	40.0 ^b^	41.5 ^b^	54.7 ^c^	41.0
Education	Lower or middle	20.6 ^a^	29.9 ^b^	31.4 ^b^	31.3 ^b^	28.6
	Higher	37.5 ^a^	42.6 ^a^	38.1 ^a^	42.1 ^a^	39.9
	University	41.9 ^a^	27.5 ^b^	30.6 ^b^	26.6 ^b^	31.5
Employment	Student	7.7 ^a^	7.2 ^a^	4.0 ^b^	2.7 ^b^	5.4
	Full-time employed	73.5 ^a,b^	69.4 ^b,c^	77.1 ^a^	67.0 ^c^	71.8
	Part-time employed	16.1 ^a^	18.5 ^a^	14.1 ^a^	23.8 ^b^	18.0
	Unemployed	2.6 ^a^	4.9 ^a,b^	4.8 ^a,b^	6.6 ^b^	4.7
Distance Event	5 km	13.2 ^a^	11.0 ^a^	3.7 ^b^	5.1 ^b^	7.9
	10 km	19.7 ^a^	17.3 ^a^	13.1 ^b^	16.0 ^a,b^	16.2
	21.1 km	50.9 ^a^	55.8 ^a,b^	58.0 ^b^	52.8 ^a,b^	55.1
	42.2 km	16.1 ^a^	15.9 ^a^	25.2 ^b^	26.1 ^b^	20.9
Main sport	Running	50.9 ^a^	72.4 ^b^	82.8 ^c^	85.6 ^c^	73.5
	Other sport	49.1 ^a^	27.6 ^b^	17.2 ^c^	14.4 ^c^	26.5
Experience	<1 year	22.9 ^a^	16.7 ^b^	10.1 ^c^	6.2 ^d^	13.7
	1–5 years	44.3 ^a^	40.9 ^a^	40.9 ^a^	34.7 ^b^	40.5
	>5 years	32.8 ^a^	42.5 ^b^	49.0 ^c^	59.1 ^d^	45.8
Training Distance	≤5 km/session	14.2 ^a^	11.5 ^a^	3.3 ^b^	3.4 ^b^	7.9
	6–10 km/session	48.3 ^a^	42.0 ^b^	33.8 ^c^	35.4 ^c^	39.6
	11–15 km/session	32.5 ^a^	37.4 ^a^	47.8 ^b^	48.6 ^b^	41.8
	≥16 km/session	5.0 ^a^	9.1 ^b^	15.1 ^c^	12.6 ^c^	10.7
Training frequency	≤1×/week	45.6 ^a^	29.6 ^b^	18.0 ^c^	14.7 ^c^	26.5
	2×/week	38.3 ^a^	41.5 ^a^	36.2 ^a^	40.2 ^a^	39.1
	≥3×/week	16.1 ^a^	28.9 ^b^	45.8 ^c^	45.0 ^c^	34.4
Event participation	1×/year	40.8 ^a^	25.6 ^b^	17.1 ^c^	19.2 ^c^	24.9
	2–4×/year	45.8 ^a^	48.3 ^a^	43.8 ^a^	45.0 ^a^	45.6
	≥5×/year	13.4 ^a^	26.1 ^b^	39.1 ^c^	35.8 ^c^	29.6
Running context	Individual	74.4 ^a^	44.6 ^b^	61.9 ^c^	54.6 ^d^	58.8
	Friends, colleagues, small groups	20.1 ^a^	32.1 ^b^	20.6 ^a^	23.0 ^a^	23.4
	Clubs	5.5 ^a^	23.3 ^b^	17.5 ^c^	22.4 ^b^	17.8

Chi-square with Bonferroni adjustment. Superscript letters denote subsets for which respective measures in the second column do not differ significantly at the 0.05 level. For stacked column graphs of Table 3 see Figures S1–S5.

**Table 4:** Use of technology related to type of runner in percentages, tested with chi-square and Bonferroni adjustment between type of runners.

Variable	Measurement	Casual Individual	Social Competitive	Individual Competitive	Devoted	Mean
Technology use	No use	14.1 ^a^	15.5 ^a^	6.7 ^b^	12.2 ^a^	12.1
	App	41.1 ^a^	26.7 ^b^	25.3 ^b,c^	20.7 ^c^	28.4
	Sports watch	44.8 ^a^	57.8 ^b^	68.0 ^c^	67.1 ^c^	59.5

Chi-square with Bonferroni adjustment; superscript letters denote subsets for which respective measures in the second column do not differ significantly at the 0.05 level.

**Table 5:** App users related to type of runners in percentages, tested with chi-square and Bonferroni adjustment between types.

Variable	Measurement	Casual Individual	Social Competitive	Individual Competitive	Devoted	Average
What do you monitor?	Distance	98.9	98.5	98.4	95.9	98.2
	Time	97.8	97.0	96.5	93.5	96.6
	Speed	95.1	93.7	95.7	91.2	94.2
	Heart rate	9.9	10.0	7.0	8.8	9.1
	Other (cadence, kcal)	3.0 a	5.6 ^a,b^	5.9 ^a,b^	9.4 b	5.4
What do you do with the data?	Nothing	7.1	6.7	4.7	7.1	6.4
	Review session after the run	81.0	79.9	82.0	76.5	80.3
	Monitor data over time	53.6 ^a^	54.3 ^a^	65.6 ^b^	55.3 ^a,b^	56.9
	Use data to adapt training	9.6	10.0	14.8	14.1	11.7

Chi-square with Bonferroni adjustment; superscript letters denote subsets for which respective measures in the second column do not differ significantly at the 0.05 level. For numbers without a letter, no significant differences were found between types of runners for that specific measurement.

**Table 6:** Sports watch users related to types of runners in percentages, tested with chi-square and Bonferroni adjustment between types.

Variable	Measurement	Casual Individual	Social Competitive	Individual Competitive	Devoted	Average
What do you monitor?	Distance	87.4 ^a^	88.0 ^a,b^	92.3 ^b^	91.3 ^a,b^	90.0
	Time	96.5	95.2	96.2	96.4	96.0
	Speed ^1^	85.9 ^a^	87.3 ^a,b^	91.0 ^b^	88.4 ^a,b^	85.5
	Heart rate	68.8 ^a,b^	65.2 ^b^	72.1 ^a^	66.1 ^a,b^	68.2
	Other (cadence, kcal)	8.6	6.7	10.6	8.9	8.8
What do you do with the data?	Nothing ^2^	6.8 ^a^	7.4 ^a^	4.2 ^b^	4.9 ^b^	5.7
	Review the session after the run	77.1	78.2	75.7	78.6	77.3
	Monitor data over time	54.2 ^a^	52.0 ^a^	65.3 ^b^	52.1 ^a^	56.6
	Use data to adapt training	20.4 ^a,b^	15.1 ^b^	29.7 ^c^	22.0 ^a^	22.3

Chi-square with Bonferroni adjustment; superscript letters denote subsets for which respective measures in the second column do not differ significantly at the 0.05 level. For numbers without a letter, no significant differences were found between types of runners for that specific measurement. ^1^ *p* = 0.051, ^2^ *p* = 0.059

**Table 7:** Non-users related to types of runners in percentages, tested with chi-square and Bonferroni adjustment between types.

Variable	Measurement	Casual Individual	Social Competitive	Individual Competitive	Devoted	Average
Reasons for not using technology	Running with phone/watch is ignorant	32.8	32.1	37.7	35.0	33.8
	No added value	45.6 ^a,b^	32.1 ^b^	32.4 ^a,b^	51.5 ^a^	40.2
	No need to	36.8	33.3	42.6	41.0	37.4
	Does not fit my running needs	28.0 ^a,b^	17.3 ^b^	20.6 ^a,b^	32.0 ^a^	24.1

Chi-square with Bonferroni adjustment; superscript letters denote subsets for which respective measures in the second column do not differ significantly at the 0.05 level. For numbers without a letter, no significant differences were found between types of runners for that specific measurement.

## Supplementary Materials

The following are available online at https://www.mdpi.com/1660-4601/17/7/2276/s1, File S1: questionnaire ERS2016, Figure S1: staked column graphs independent variables for whole sample, Figure S2: staked column graphs independent variables for individual competitive runners, Figure S2: staked column graphs independent variables for individual competitive runners, Figure S3: staked column graphs independent variables for casual individual runners, Figure S4: staked column graphs independent variables for social competitive runners, Figure S5: staked column graphs independent variables for devoted runners.
