# Supplementary material for: Understanding Different Types of Recreational Runners and How They Use Running-Related Technology
Source: Int J Environ Res Public Health. 2020 Mar 27;17(7):2276. doi: 10.3390/ijerph17072276 (PMC7177805; doi:10.3390/ijerph17072276)
Supplement: Supplementary file 1 [file ijerph-17-02276-s001.zip › Supp Files Rev3/S1 Questionnaire ERS2016.pdf]

Questionnaire ERS2016

SECTION 1 AIOs running

Please respond to the following statements by checking the box that best reflects your opinion on each of the following items.

	Strongly disagree.	Disagree.	Neutral.	Agree	Strongly agree
The possibility to run on your own is an important reason to be involved in running[4]					
The possibility to run at any time is an important reason to be involved in running[4]					
The possibility to run in your own environment is an important reason to be involved in running[4]					
Running is a social sport[3]					
Running is appealing for both men and women					
Running is a competitive sport[6]					
Running is appealing for both youngsters and the elderly					
Running is an exciting sport[3]					
Running is for those who persevere					
Running is easy to fit into daily life					
Running is good for your condition[1]					
Running is good for your health[1]					
Running is an individual sport					
Running gives you energy[1]					
Running is good for your mental recovery[1]					
I am proud to be a runner[3]					
I feel connected with other runners[3]					
Running is a performance sport[6]					
I consider myself to be a real runner[3]					

To which extent do you agree that the following items are a reason for you to quit running? Please respond by checking the box that best reflects your opinion.

	Strongly disagree.	Disagree.	Neutral.	Agree	Strongly agree
My running partners quit running[2]	○	○	○	○	○
My running group falls apart[2]	○	○	○	○	○
My trainer / coach is leaving[2]	○	○	○	○	○
Increasing financial costs involved in running	○	○	○	○	○
Preference for another sport[5]	○	○	○	○	○
Reduction of leisure time[5]	○	○	○	○	○
Tired of running[5]	○	○	○	○	○
Physical constraints or injuries[5]	○	○	○	○	○

Continue to SECTION 2

SECTION 2 technology use

Did you use a monitoring device, sports watch or smartphone application during running in the last 12 months?

- ◯ Yes, I used a sports watch (go to SECTION 2 sport watch)
- ◯ Yes, I used a smartphone application (go to SECTION 2 app)
- ◯ No, I did not use any sports watch or smartphone application (go to SECTION 2 no use)

SECTION 2 sports watch

You stated to use a sports watch during running in the last 12 months.

Which parameters do you monitor with your sports watch? (multiple answers possible)

- Distance
- Time
- Speed
- Heart rate
- Other, please specify

What do you do with the data collected from your watch? (multiple answers possible)

- Nothing
- I use the data to review my session after the run
- I use this data to monitor my progress overtime
- I use this data to adapt my training / running schedule

Continue to SECTION 3

SECTION 2 app

You stated to use a smartphone application (app) during running in the last 12 months.

Which parameters do you monitor with your app? (multiple answers possible)

- ◯ Distance
- ◯ Time
- ◯ Speed
- ◯ Heart rate
- ◯ Other, please specify

What do you do with the data collected from your app? (multiple answers possible)

- ◯ Nothing
- ◯ I use the data to review my session after the run
- ◯ I use this data to monitor my progress overtime
- ◯ I use this data to adapt my training / running schedule

Continue to SECTION 3

SECTION 2 no use

You stated not to use any sports watch or smartphone application during running. Why not? (multiple answers possible)

- Running with a smartphone/watch is ignorant
- It has no added value
- There is no need for me to use it
- It doesn't fit with my running experience

Continue to SECTION 3

SECTION 3 Running habits and Socio-demographics

Which distance did you run during the event?

- 5km
- 10km
- Half Marathon
- Marathon

Is running your main sport?

- No
- Yes

When did you start running?

- less than 1 year ago
- 1 to 3 years ago
- 4 to 5 years ago
- More than 5 years ago

On average, how many kilometers per training session do you run?

- 0-5 km
- 6-10 km
- 11-15 km
- 16 or more km

How often do you run?

- Once a week or less
- 2 times a week
- 3 times a week or more

How often did you participate in running events in the last 12 months?

- This event was my only running event
- 2-4 times a year
- 5 or more times a year

In which setting do you normally run? (choose the best fit)

- ◯ Mainly or solely on my own
- ◯ Mainly or solely with friends, colleagues, small groups
- ◯ Mainly or solely with a running club

What is your age?

[ ]

What is your gender?

- ◯ Male
- ◯ Female

What is your employment status? Are you currently?

- ◯ A student
- ◯ Full-time employed
- ◯ Part-time employed
- ◯ Not employed

What is the highest degree or level of school you have completed?

- ◯ Lower or middle education
- ◯ Higher education
- ◯ University
