# Supplementary material for: Understanding Different Types of Recreational Runners and How They Use Running-Related Technology
Source: Int J Environ Res Public Health. 2020 Mar 27;17(7):2276. doi: 10.3390/ijerph17072276 (PMC7177805; doi:10.3390/ijerph17072276)
Supplement: Supplementary file 1 [file ijerph-17-02276-s001.zip › Supp Files Rev3/Figure S2.pdf]

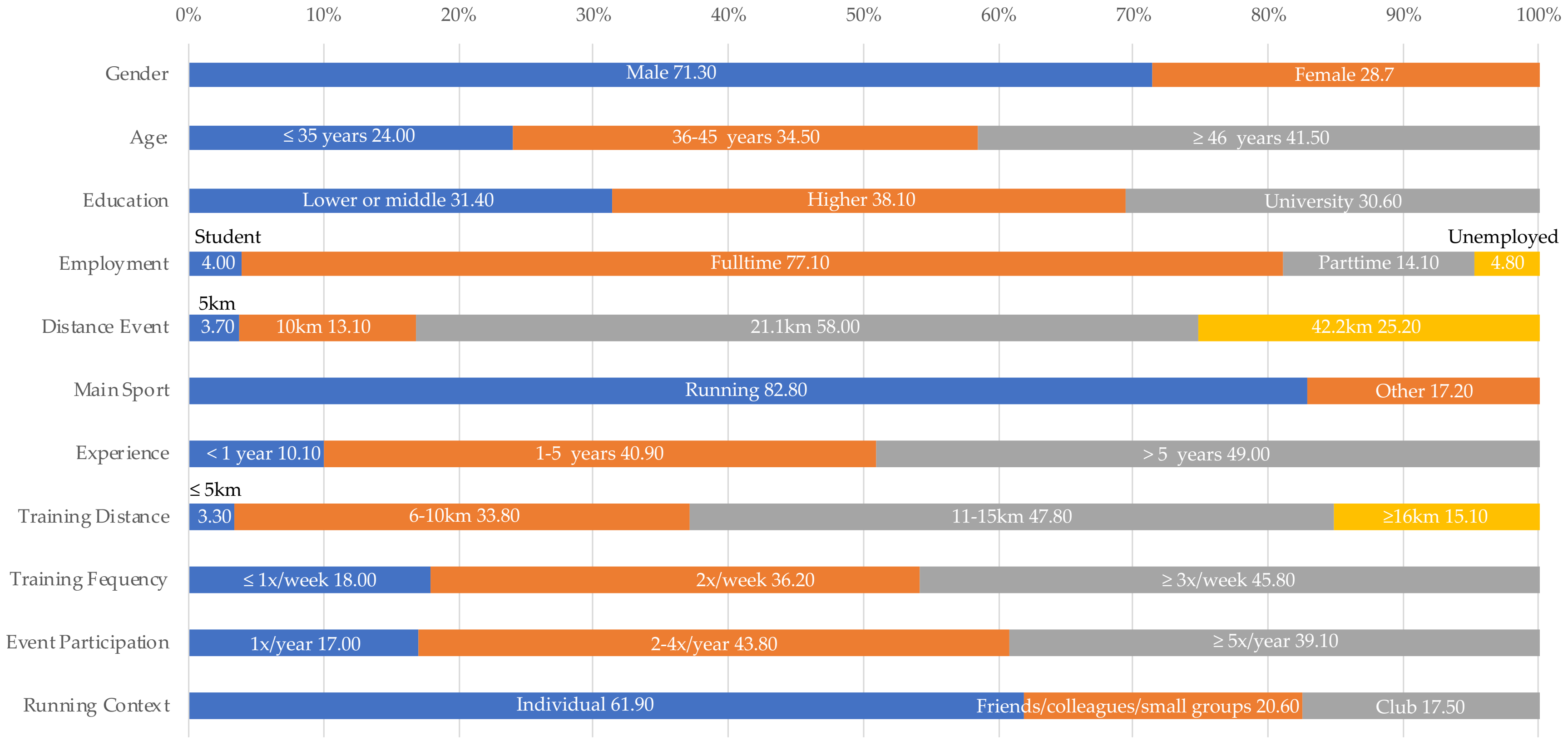

Figure S2. Summary of independent variables for Individual Competitive Runners in percentages (N = 1012).
