# Supplementary figures and images for: Understanding Different Types of Recreational Runners and How They Use Running-Related Technology
Source: Int J Environ Res Public Health. 2020 Mar 27;17(7):2276. doi: 10.3390/ijerph17072276 (PMC7177805; doi:10.3390/ijerph17072276)

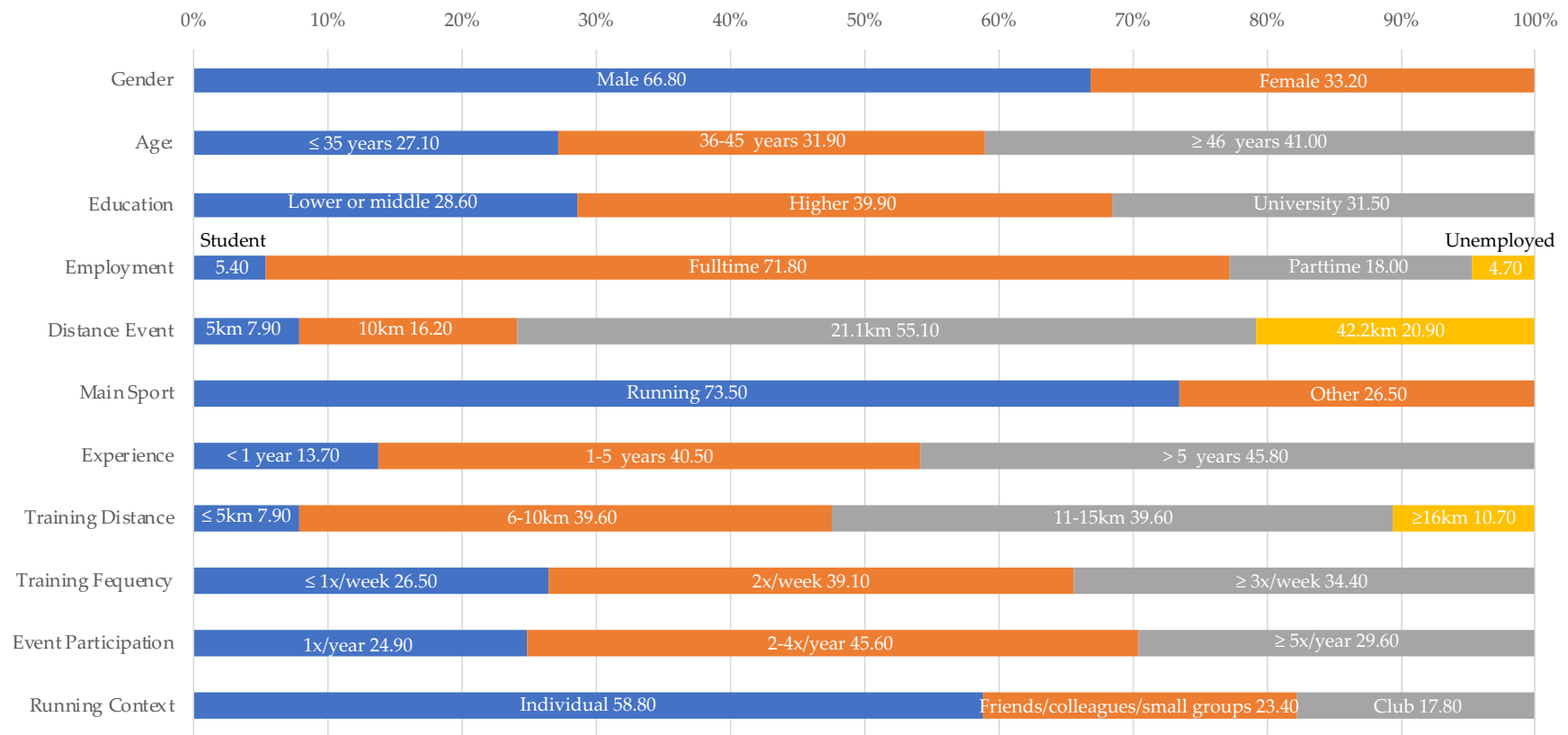

Figure S1. Summary of independent variables for all runners in percentages (N = 3727).

Supplement: Supplementary file 1 [file ijerph-17-02276-s001.zip › Supp Files Rev3/Figure S1.pdf]

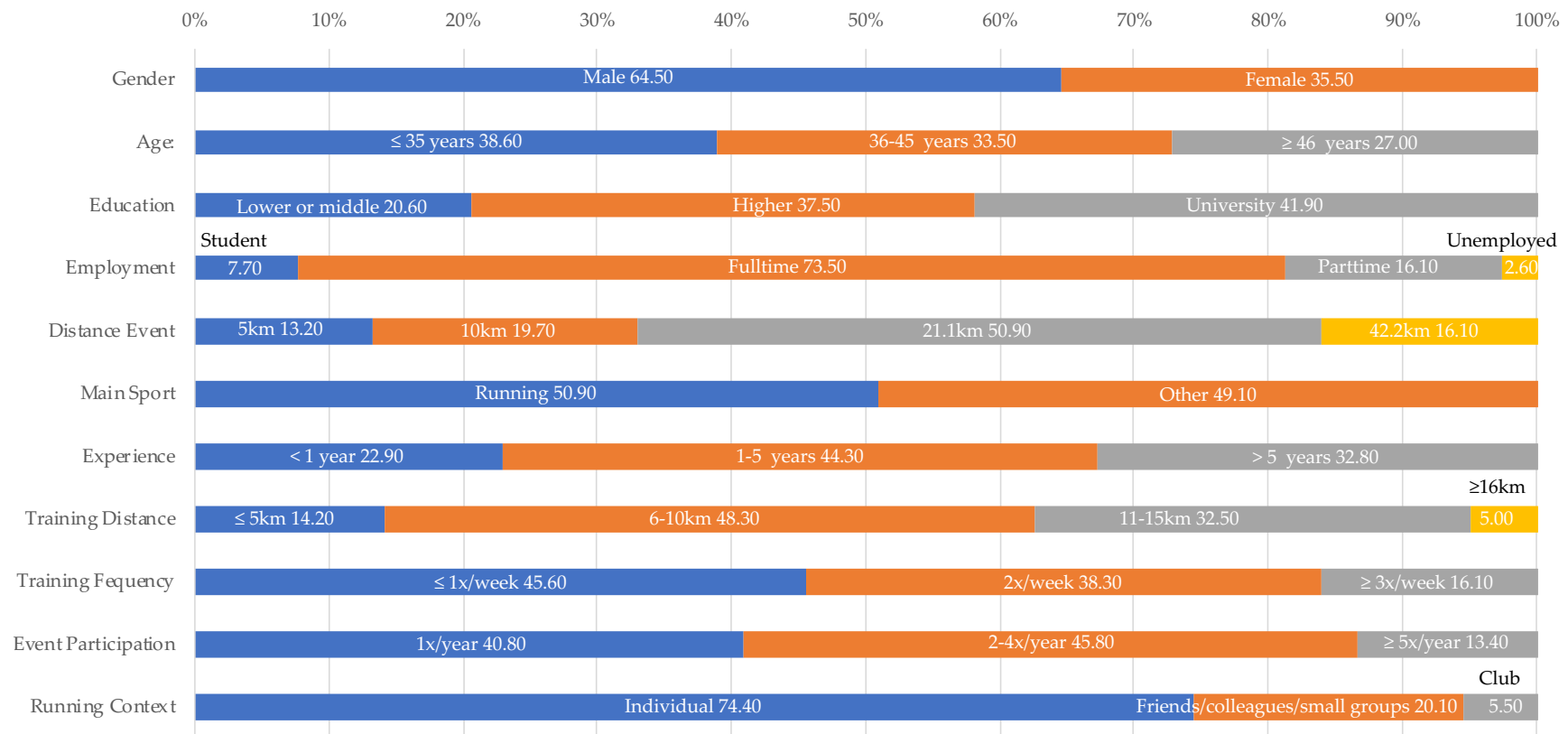

Figure S3. Summary of independent variables for Casual Individual Runners in percentages (N = 886).

Supplement: Supplementary file 1 [file ijerph-17-02276-s001.zip › Supp Files Rev3/Figure S3.pdf]

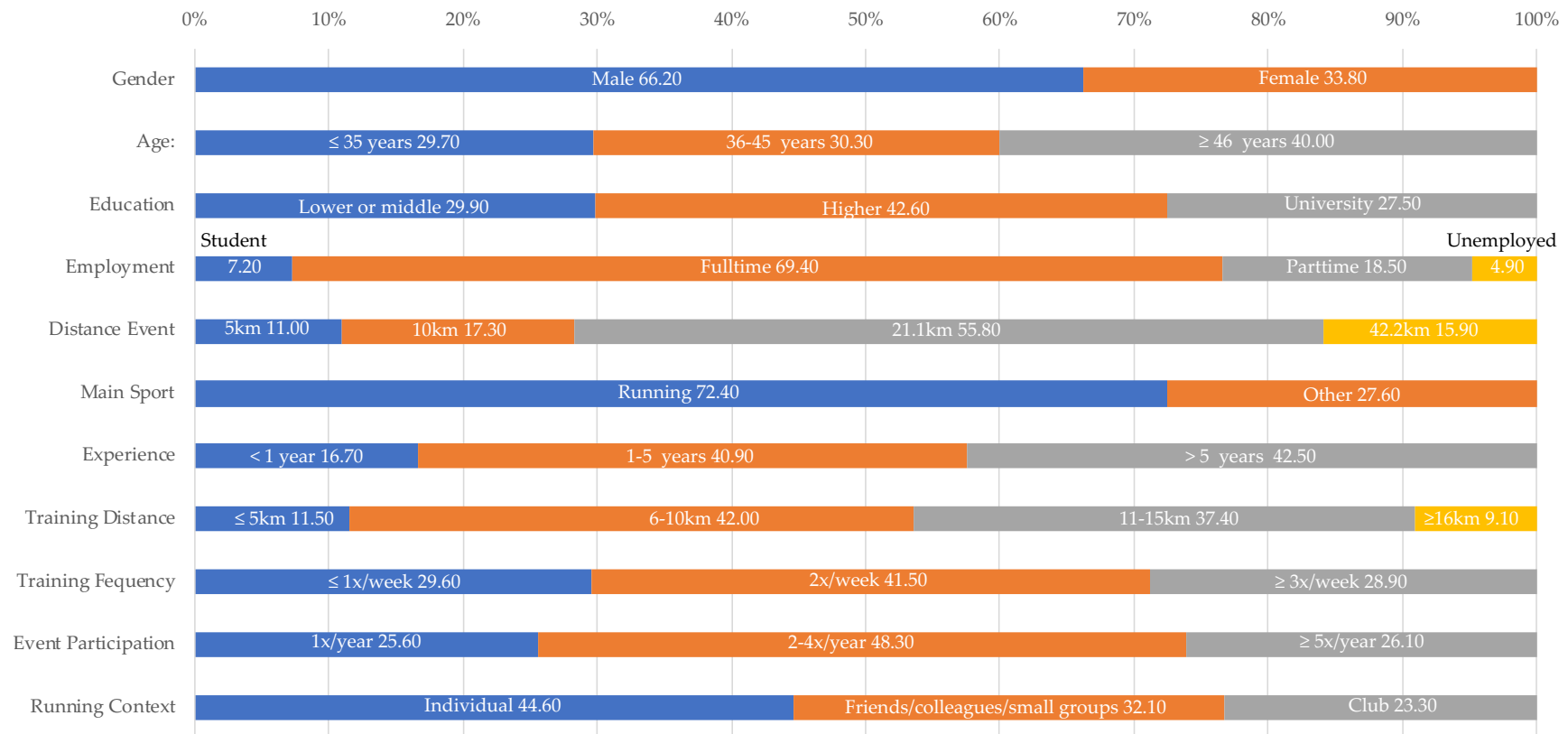

Figure S4. Summary of independent variables for Social Competitive Runners in percentages (N = 1008).

Supplement: Supplementary file 1 [file ijerph-17-02276-s001.zip › Supp Files Rev3/Figure S4.pdf]

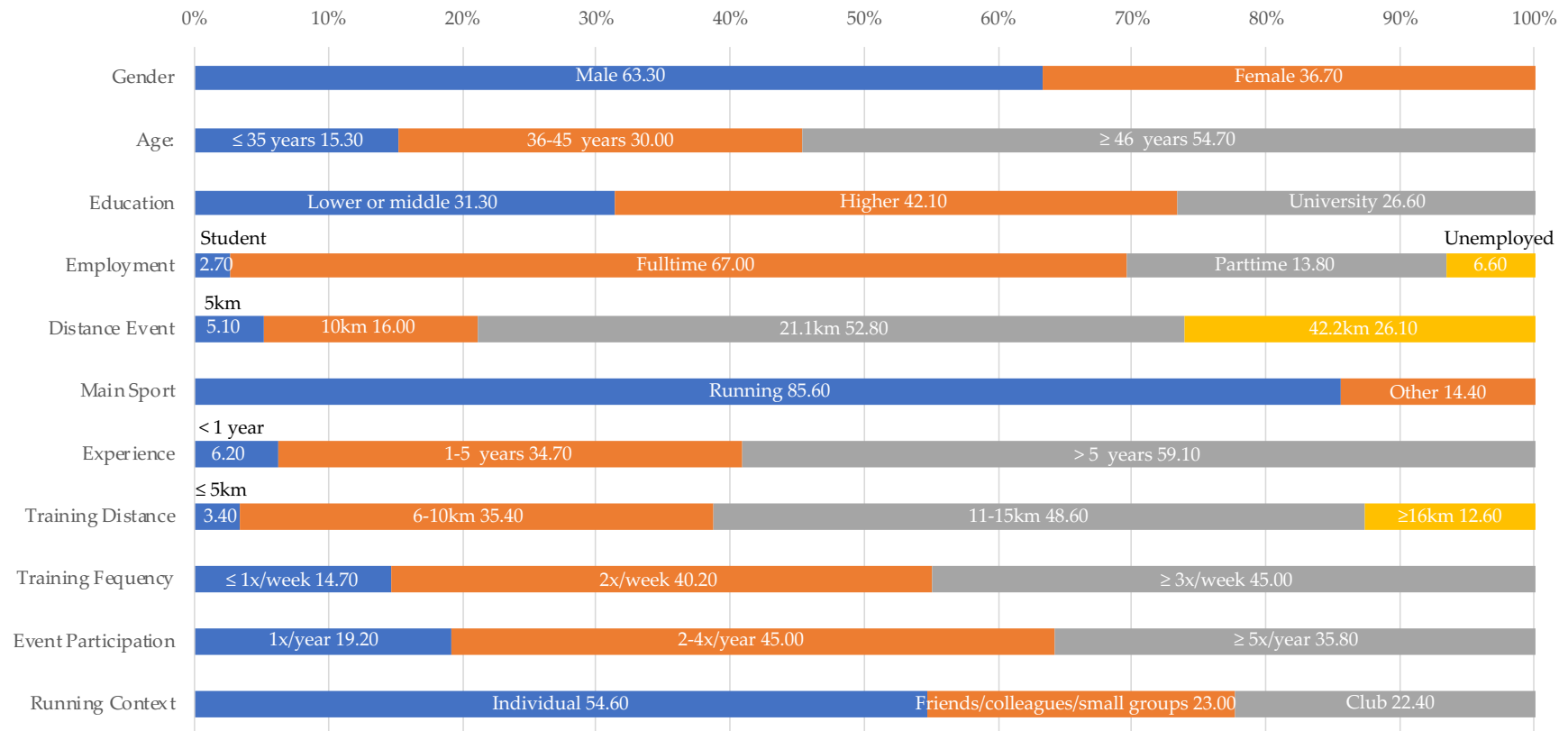

Figure S5. Summary of independent variables for Devoted Runners in percentages (N = 821).

Supplement: Supplementary file 1 [file ijerph-17-02276-s001.zip › Supp Files Rev3/Figure S5.pdf]
